# Effectiveness of a cluster management strategy on maternal and neonatal outcomes in high-risk pregnancies with hypertensive disorders: a retrospective cohort study

**DOI:** 10.3389/fgwh.2026.1776715

**Published:** 2026-06-17

**Authors:** Longhua Zhang, Yilin Wang, Lingling Jiang, Ying Gu, Xiaokang Deng

**Affiliations:** 1 Wuxi Maternity and Child Health Care Hospital, Affiliated Women’s Hospital of Jiangnan University, Wuxi, Jiangsu Province, China; 2 Department of Obstetrics, Affiliated Hospital 2 of Nantong University, Nantong City, Jiangsu Province, China; 3 Southeast University Affiliated Nantong First People’s Hospital, Nantong City, Jiangsu Province, China; 4 School of Biotechnology, Jiangnan University, Wuxi, Jiangsu Province, China

**Keywords:** aspirin prophylaxis, care bundle, cluster management, gestational hypertension, maternal-fetal outcomes

## Abstract

**Background:**

Hypertensive disorders of pregnancy (HDP) are a leading cause of maternal and perinatal morbidity. Traditional management often lacks systematic integration of preventive and therapeutic measures. This study evaluated the efficacy of a cluster management strategy in high-risk HDP pregnancies.

**Methods:**

A retrospective cohort study was conducted on 103 pregnant women with HDP risk factors admitted between January 2024 and February 2025. Patients were categorized into an Observation Group (cluster management, *n* = 55) and a Control Group (routine care, *n* = 48). The cluster management strategy integrated early aspirin prophylaxis, dynamic blood pressure thresholds, and precise volume management.

**Findings:**

Despite having significantly higher pre-pregnancy BMI (26.64 ± 4.33 vs. 24.14 ± 4.92 kg/m^2^, *P* < 0.05), the Observation Group exhibited a delayed onset of proteinuria compared to the Control Group (34.67 ± 5.06 vs. 31.50 ± 7.69 weeks, *P* = 0.021). The incidence of pleural/peritoneal effusion (25.5% vs. 52.1%) and hypoproteinemia (18.2% vs. 37.5%) was significantly reduced in the Observation Group (*P* < 0.05). Crucially, the mean gestational age at delivery was prolonged to 37.51 ± 1.94 weeks in the Observation Group, compared to 34.57 ± 2.90 weeks in the Control Group (*P* < 0.001). Consequently, neonatal outcomes improved, evidenced by lower NICU admission rates (52.7% vs. 85.1%) and reduced hospitalization costs (*P* < 0.001).

**Conclusions:**

A cluster management strategy based on early pharmacological prevention and comprehensive monitoring effectively delays disease progression and prolongs expectant management duration in high-risk pregnancies, offering significant clinical and health-economic benefits.

## Introduction

Hypertensive disorders of pregnancy (HDP), particularly preeclampsia, remain a major global health challenge, contributing significantly to maternal and perinatal mortality. The pathophysiology involves placental malperfusion and systemic endothelial dysfunction, often necessitating preterm delivery to preserve maternal safety (1, 2). While guidelines from organizations such as ACOG and ISSHP provide frameworks for management, clinical implementation often suffers from fragmentation and delayed intervention.

Recent evidence, notably the ASPRE trial, has established the efficacy of low-dose aspirin in preventing preterm preeclampsia when initiated in the first trimester (3). However, the translation of pharmacological prevention into a holistic “Cluster Management” (or Care Bundle) approach-integrating risk stratification, hemodynamic monitoring, and multidisciplinary collaboration — remains underexplored in real-world clinical settings.

This study aims to evaluate a comprehensive cluster management protocol centered on “Early Identification, Prophylactic Medication, and Dynamic Warning.” We hypothesized that this structured approach could mitigate disease progression and improve outcomes in a cohort of high-risk pregnant women, specifically those with metabolic risk factors.

## STAR methods

### Resource availability

#### Lead contact

Further information and requests for resources should be directed to and will be fulfilled by the Lead Contact, [Xiaokang Deng] ([xiaokang@jiangnan.edu.cn]).

#### Materials availability

This study did not generate new unique reagents.

#### Data and code availability

All data reported in this paper will be shared by the lead contact upon request. This paper does not report original code. Any additional information required to reanalyze the data reported in this paper is available from the lead contact upon request.

### Experimental model and subject details

#### Study design and participants

This retrospective cohort study was conducted at the Department of Obstetrics, [*Wuxi Maternity and Child Health Care Hospital*], China. The study period spanned from January 2024 to February 2025. We screened pregnant women with high-risk factors for hypertensive disorders of pregnancy (HDP) who established perinatal care files and delivered at our institution.

#### Inclusion and exclusion criteria

Inclusion criteria: (1) Presence of at least one high-risk factor for HDP (e.g., advanced maternal age ≧ 35 years, pre-pregnancy obesity [BMI ≧ kg/m^2^], history of chronic hypertension, or history of preeclampsia), assessed according to the Guidelines for the Diagnosis and Treatment of Hypertensive Disorders of Pregnancy (2020) (4); (2) Singleton pregnancy; (3) Availability of complete clinical data.

Exclusion criteria: (1) Confirmed fetal chromosomal abnormalities or major structural malformations; (2) Termination of pregnancy for non-medical reasons.

#### Ethical approval

The study protocol was approved by the Ethics Committee of [*Wuxi Maternity and Child Health Care Hospital*] (Approval No. [XJSLLYL2024009]). The requirement for informed consent was waived due to the retrospective nature of the study, and patient data were anonymized.

### Method details

#### Group allocation

A total of 103 eligible patients were included in the final analysis. Based on the actual management protocols received during pregnancy, participants were categorized into the Control Group (*n* = 48) and the Observation Group (*n* = 55).

#### Control group protocol (routine care)

Patients in the Control Group received standard obstetric care, which included:

1. Establishment of perinatal health records and regular prenatal examinations.
2. Routine health education regarding diet, rest, and lifestyle.
3. Pharmacological treatment initiated only upon the diagnosis of hypertension (Systolic BP ≧ 140 mmHg and/or Diastolic BP ≧ 90 mmHg). Antihypertensive agents (e.g., Labetalol or Nifedipine) were administered strictly according to physician orders. Symptomatic management upon the occurrence of complications. Of note, patients in the Control Group did not receive systematic early aspirin prophylaxis for prevention of preeclampsia or other hypertensive disorders.

#### Observation group protocol (cluster management strategy)

In addition to routine care, the Observation Group received a comprehensive “Cluster Management” intervention characterized by four core components:

1. Early Pharmacological Prophylaxis: Following FIGO guidelines and national expert consensus, patients identified as high-risk during first-trimester screening initiated low-dose aspirin therapy (75–100 mg/day) between 12 and 16 gestational weeks. This prophylactic regimen was continued until 34–36 gestational weeks to improve placental perfusion (4, 5).
2. Dynamic Blood Pressure Surveillance and Warning System: A dynamic monitoring system was established, integrating home blood pressure monitoring with clinical oversight. Patients were trained on standardized measurement techniques. A “Warning Threshold” was set at SBP ≧ 140 mmHg or DBP≧ 90 mmHg. Any trigger of this threshold prompted immediate medical evaluation and early initiation or adjustment of antihypertensive medication to prevent severe hemodynamic fluctuations.
3. Precise Volume Management and Target Organ Protection: To address the pathophysiology of endothelial leakage and hypoproteinemia in HDP, rigorous surveillance of serum albumin levels and 24-hour urinary protein was implemented. Nutritional interventions (high-protein diet) or intravenous albumin supplementation were administered promptly to patients showing trends of hypoproteinemia (Albumin < 30 g/L) to maintain colloid osmotic pressure and prevent pleural/peritoneal effusion.
4. Optimization of Delivery Timing via MDT: A Multidisciplinary Team (MDT)-comprising senior obstetricians, neonatologists, and anesthesiologists-conducted dynamic assessments of maternal-fetal status. The primary goal was to safely extend the expectant management duration, targeting a delivery gestational age of ≧ 37 weeks to minimize prematurity-related complications.

#### Outcome measures

##### Disease progression indicators

Gestational age at first onset of proteinuria, gestational age at initiation of antihypertensive medication, and total aspirin dosage. Maternal Morbidity: Incidence of severe complications, including hypoproteinemia (Albumin < 30 g/L), pleural/peritoneal effusion, HELLP syndrome, and hypertensive retinopathy.

##### Perinatal outcomes

Gestational age at delivery, mode of delivery, neonatal Apgar scores (1-min and 5-min), Neonatal Intensive Care Unit (NICU) admission rate, length of neonatal hospital stay, and hospitalization costs.

### Quantification and statistical analysis

Data analysis was performed using SPSS software, version 26.0 (IBM Corp., Armonk, NY, USA). Continuous variables were tested for normality using the Kolmogorov–Smirnov test. Normally distributed data were expressed as mean ± standard deviation (x ± s) and compared using the independent Student’s t-test. Categorical variables were expressed as frequencies and percentages [n (%)] and compared using the Chi-square test (x 2) or Fisher’s exact test, as appropriate. A two-sided *P*-value < 0.05 was considered statistically significant.

## Results

### Baseline characteristics and risk profile

A total of 103 patients were included (Observation: *n* = 55; Control: *n* = 48). There were no significant differences in maternal age, parity, or mode of conception between the two groups (*P* > 0.05). Notably, the Observation Group presented with a significantly higher metabolic risk profile at baseline, with a pre-pregnancy Body Mass Index (BMI) of 26.64 ± 4.33 kg/m^2^ compared to 24.14 ± 4.92 kg/m^2^ in the Control Group (*P* < 0.05) (Table 1).

**Table 1:** Baseline characteristics and risk profile ($\bar{x}$±s).

Variables	Observation group (*n* = 55)	Control group (*n* = 48)	t/x2	*P*-value
Maternal Age (years)	33.09 ± 5.14	34.75 ± 5.50	t = −1.58	0.119
Height (cm)	161.65 ± 5.31	160.27 ± 5.80	t = 1.26	0.212
Pre-pregnancy Weight (kg)	69.87 ± 12.86	62. 12 ± 14.10	t = 2.82	0.006
Pre-pregnancy BMI (kg/m2)	26.64 ± 4.33	24. 14 ± 4.92	t = 2.66	0.01
Gravidity (n)	2.35 ± 1.51	2.35 ± 1.44	t = −0.03	0.976
Parity (n)	0.44 ± 0.63	0.54 ± 0.77	t = −0.75	0.454
Mode of conception				
natural conception	44 (80.0%)	37 (86.0%)	x2 = 0.61	0.606
assisted reproduction	11 (20.0%)	6 (14.0%)		

### Comparison of pharmacological intervention and disease progression

The Observation Group demonstrated high compliance with the aspirin prophylaxis regimen, with a mean daily dosage of 79.09 ± 25.80 mg, which was significantly higher than that of the Control Group (*P* < 0.001). The average daily aspirin dosage observed in the control group was lower (7.81 ± 23.72 milligrams), indicating an occasional, non-systematic preventive use situation. Regarding disease control, the Observation Group initiated oral antihypertensive therapy at a mean gestational age of 25.1 ± 13.2 weeks, significantly earlier than the Control Group (30.3 ± 8.6 weeks, *P* = 0.023),indicating successful implementation of early intervention. Notably, despite having a higher baseline BMI, the Observation Group exhibited a delayed onset of proteinuria.The mean gestational age at the first detection of proteinuria was 34.67 ± 5.06 weeks in the Observation Group, significantly later than 31.50 ± 7.69 weeks in the Control Group (t = 2.35, *P* = 0.021).This suggests that the cluster management strategy effective in delaying the occurrence of renal impairment (Table 2).

**Table 2:** Pharmacological intervention and disease progression ($\bar{x}$±s).

Variables	Observation group* (*n* = 55)	Control group* (*n* = 48)	t/x2	*P*-value
Aspirin Dosage (mg/day)	79.09 ± 25.80	7.81 ± 23.72	t = 14.60	<0.001
Onset of Proteinuria (weeks)	34.67 ± 5.06	31.50 ± 7.69	t = 2.35	0.021
Number of confirmed cases of preeclampsia (n)	45 (83.3%)	47 (95.9%)	x2 = 0.0808	0.0548
Diagnosis of Preeclampsia (weeks)	34.80 ± 5.06	33.36 ± 3.59	t = 1.57	0.12
BMI at diagnosis (kg/m^2^)	31.70 ± 4.10	29.04 ± 7.98	t = 2.08	0.041

* The few patients in the Control Group who took aspirin did so for other reasons (e.g., prior thrombophilia) and at irregular, low doses. The Control Group did not receive systematic aspirin prophylaxis.

### Comparison of maternal severe complications

Cluster management significantly mitigated pathological changes associated with vascular endothelial injury and leakage. The incidence of pleural/peritoneal effusion in the Observation Group was 25.5% (14/55), significantly lower than the 52.1% (25/48) observed in the Control Group (*P* = 0.010). Similarly, the incidence of hypoproteinemia (Albumin < 30 g/L) was significantly reduced in the Observation Group compared to the Control Group (18.2% vs. 37.5%, *P* = 0.048). No statistically significant differences were found between the two groups regarding the incidence of hypertensive retinopathy or HELLP syndrome (*P* > 0.05) (Table 3).

**Table 3:** Maternal morbidity and complications ($\bar{x}$±s).

Variables	Observation group (*n* = 55)	Control group (*n* = 48)	t/x2	*P*-value
Start of Antihypertensive Therapy (weeks)	25. 1 ± 13.2	30.3 ± 8.6	t = −2.31	0.023
Add antihypertensive drugs (weeks)	33.3 ± 5.0	30.3 ± 8.8	t = 1.77	0.082
Pleural/Peritoneal Effusion [*n* (%)]	14 (25.5%)	25 (52.1%)	x2 = 6.63	0.01
Hypoproteinemia (Alb < 30 g/L) [*n* (%)]	10 (18.2%)	18 (37.5%)	x2 = 3.91	0.048
Retinopathy [*n* (%)]	2 (3.6%)	2 (4.2%)		1
FGR [*n* (%)]	5 (9.1%)	9 (18.8%)	x2 = 1.30	0.255
Abnormal S/D ratio [*n* (%)]	7 (12.7%)	11 (22.9%)	x2 = 1.21	0.272

### Comparison of delivery outcomes

There was no statistically significant difference in the cesarean section rate between the two groups (86.3% vs. 87.5%, *P* > 0.05). However, a substantial improvement was observed in the timing of delivery. The mean gestational age at delivery in the Observation Group reached 37.51 ± 1.94 weeks, essentially achieving term delivery. In contrast, the mean gestational age in the Control Group was only 34.57 ± 2.90 weeks. The Observation Group prolonged the pregnancy by nearly 3 weeks compared to the Control Group, a difference that was statistically significant(*P* < 0.001) (Table 4).

**Table 4:** Comparison of delivery outcomes ($\bar{x}$±s).

Variables	Observation group (*n* = 55)	Control group (*n* = 48)	t/x2	*P*-value
Delivery week (weeks)	37.51 ± 1.94	34.57 ± 2.90	t = 5.88	<0.001
Mode of delivery [cesarean section, *n* (%)]	44/51 (86.3%)	42/48 (87.5%)	x2 = 0.00	1

### Perinatal outcomes and health economic evaluation

Neonatal outcomes were superior in the Observation Group, with 1-minute and 5-minute Apgar scores significantly higher than those in the Control Group (*P* < 0.05). The rate of admission to the Neonatal Intensive Care Unit (NICU) was 52.7% in the Observation Group, significantly lower than 85.1% in the Control Group (*P* = 0.001). Furthermore, for neonates requiring hospitalization, the Observation Group had a significantly shorter mean length of stay (8.55 ± 5.61days) compared to the Control Group (20.30 ± 17.01 days, *P* < 0.001). The mean hospitalization cost for neonates in the Observation Group was 10,435 ± 7,518 CNY, significantly lower than 28,748 ± 29,608 CNY in the Control Group (*P* < 0.001), demonstrating substantial health economic benefits (Table 5).

**Table 5:** Perinatal outcomes and health economic evaluation ($\bar{x}$±s).

Variables	Observation group (*n* = 55)	Control group (*n* = 48)	t/x2	*P*-value
1-min Apgar Score	9.82 ± 0.52	9. 11 ± 1.58	t = 2.97	0.004
5-min Apgar Score	9.96 ± 0.20	9.72 ± 0.68	t = 2.30	0.025
NICU Admission [*n* (%)]	29 (52.7%)	40 (85.1%)	x2 = 10.71	0.001
NICU Length of Stay (days)	8.55 ± 5.61	20.30 ± 17.01	t = −4.07	<0.001
Hospitalization Cost (CNY)	10,434 ± 7,517	28,748 ± 29,608	t = −3.75	<0.001

## Discussion

### The “prevention paradox”: overcoming metabolic risks via early intervention

A pivotal finding of this study is the “reversal of risk” observed in the cluster management cohort. Epidemiological evidence strongly correlates elevated BMI with an increased risk and severity of preeclampsia due to chronic systemic inflammation and oxidative stress (6). In our study, the Observation Group presented with a significantly higher pre-pregnancy BMI (26.6 vs. 24.1 kg/m^2^) compared to the Control Group. Paradoxically, these high-risk patients exhibited a delayed onset of proteinuria by approximately 3.2 weeks. This counter-intuitive outcome strongly validates the efficacy of our “early blocking” strategy. Specifically, the strict adherence to low-dose aspirin (79 mg/day) initiated between 12-16 weeks was likely the decisive factor. While the ASPRE trial established the benefit of aspirin, recent meta-analyses suggest that the prophylactic effect is dose-dependent and weight-dependent (7). Our study confirms that even in a metabolically compromised population, standardized aspirin prophylaxis, when combined with intensive surveillance, can effectively remodel the placental vascular bed and delay the “tipping point” of endothelial decompensation.

### Pathophysiological basis: endothelial protection and volume management

The fundamental pathology of HDP involves widespread endothelial dysfunction, leading to increased vascular permeability, hypoproteinemia, and third-space fluid accumulation (e.g., pleural effusion). These complications are often the primary indications for iatrogenic preterm delivery (6).Our cluster management protocol integrated a unique component of “Precise Volume Management,” which is often overlooked in routine care. By dynamically monitoring serum albumin and strictly managing fluid balance, we significantly reduced the incidence of pleural/peritoneal effusion (25.5% vs. 52.1%) and hypoproteinemia (18.2% vs. 37.5%). We postulate that maintaining colloid osmotic pressure and preventing severe edema stabilized the maternal systemic condition. This “maternal stabilization” is the mechanistic key that allowed for the safe prolongation of pregnancy, transforming a potential emergency termination into a controlled, near-term delivery.

### The clinical value of prolonged gestation: From “survival” to “quality”

The ultimate goal of HDP management is to balance maternal safety with fetal maturity. Our data demonstrate that cluster management extended the mean gestational age from 34.57 weeks (late preterm) in the Control Group to 37.51 weeks (early term) in the Observation Group. This 3-week extension is clinically profound. At 34 weeks, neonates remain at risk for respiratory distress syndrome (RDS) and feeding difficulties; whereas at 37 weeks, physiological maturity is largely achieved. This shift explains the dramatic reduction in NICU admissions (52.7% vs. 85.1%) observed in our study. Unlike previous studies that focused solely on maternal blood pressure control, our multidimensional approach confirms that optimizing maternal endothelial function directly translates into improved intrauterine survival time and neonatal quality of life.

### Preeclampsia recurrence and long-term implications

Preeclampsia is not only a major cause of acute maternal and neonatal morbidity but also a strong predictor of future cardiovascular and metabolic disease, as well as recurrence in subsequent pregnancies (8). Women with a history of severe or early-onset preeclampsia have an estimated recurrence rate of approximately 15–20% in a future pregnancy, with even higher rates in those with chronic hypertension or persistent obesity (9). Our cluster management strategy, which demonstrated efficacy in prolonging gestation and reducing complications in the index pregnancy, carries potential implications for recurrence prevention. Specifically, the early initiation of low-dose aspirin and sustained hemodynamic monitoring could be reduce the risk of subsequent pregnancies. Furthermore, the BMI of our intervention group was higher, but the treatment effect was better. This indicates that the bundled management strategy might be able to overcome some unfavorable metabolic factors. This finding is particularly significant for preventing recurrent preeclampsia. Although our study did not follow up on the patients’ conditions after this pregnancy, we speculate that if the Cluster management approach is applied in future pregnancies, it is likely to reduce the recurrence rate of preeclampsia. It will be necessary to conduct multi-center prospective studies in the future to evaluate whether the cluster management program can reduce the recurrence rate of preeclampsia and improve its long-term cardiovascular health.

### Health economic implications and care standardization

In an era of rising healthcare costs, the economic viability of clinical interventions is crucial. Our study provides compelling evidence that cluster management is a cost-effective strategy. The substantial reduction in neonatal hospital stay (reduction of∼12 days) and costs (reduction of∼64%) offsets the resources required for intensive antenatal monitoring. Furthermore, the success of this retrospective cohort underscores the importance of protocol standardization. HDP management is often fragmented; our “Cluster” approach ensures that critical steps — screening, aspirin prophylaxis, BP warning, and MDT evaluation—are executed consistently. This aligns with the ISSHP’s global call for standardized care pathways to reduce maternal morbidity (1).

### Strengths and limitations

The strength of this study lies in its “real-world” applicability and the integration of a comprehensive care bundle rather than a single intervention. However, limitations exist. First, the retrospective design introduces potential selection bias, although the higher baseline risk in the intervention group suggests that our results may actually underestimate the efficacy of the protocol. Second, the sample size was relatively small and confined to a single center. Future multi-center randomized controlled trials (RCTs) are warranted to validate these findings and to explore the optimal aspirin dosage for specific BMI categories.

## Conclusion

In this retrospective cohort study, a cluster management strategy integrating early aspirin prophylaxis, dynamic blood pressure monitoring, precise volume management, and multidisciplinary team evaluation significantly delayed disease progression, prolonged gestational age at delivery (from 34.57 weeks to 37.51 weeks), and improved neonatal outcomes (reducing NICU admission rates from 85.1% to 52.7%). Despite a higher baseline metabolic risk (higher BMI) in the intervention group, the strategy effectively reduced maternal complications such as pleural/peritoneal effusion and hypoproteinemia, and substantially lowered NICU admission rates and hospitalization costs. These findings support the implementation of standardized cluster management protocols for high-risk pregnant women with hypertensive disorders of pregnancy. Future multicenter randomized controlled trials are needed to further validate these results and optimize aspirin dosing based on BMI categories.

## Data Availability

The raw data supporting the conclusions of this article will be made available by the authors, without undue reservation.
